# Vocational rehabilitation services for patients with cancer: design of a feasibility study incorporating a pilot randomised controlled trial among women with breast cancer following surgery

**DOI:** 10.1186/1745-6215-12-89

**Published:** 2011-03-30

**Authors:** Richard G Kyle, Bill Culbard, Josie Evans, Nicola M Gray, Dolapo Ayansina, Gill Hubbard

**Affiliations:** 1 Cancer Care Research Centre, School of Nursing, Midwifery and Health, University of Stirling, Stirling, UK; 2 School of Nursing, Midwifery and Health, University of Stirling, Stirling, UK; 3 Centre of Academic Primary Care, Division of Applied Health Sciences, School of Medicine and Dentistry, University of Aberdeen, UK; 4 Division of Applied Health Sciences, School of Medicine and Dentistry, University of Aberdeen, UK

## Abstract

**Background:**

Due to improvements in cancer survival the number of people of working age living with cancer across Europe is likely to increase. UK governments have made commitments to reduce the number of working days lost to ill-health and to improve access to vocational rehabilitation (VR) services. Return to work for people with cancer has been identified as a priority. However, there are few services to support people to remain in or return to work after cancer and no associated trials to assess their impact. A pilot randomised controlled trial among women with breast cancer has been designed to assess the feasibility of a larger definitive trial of VR services for people with cancer.

**Methods:**

Patients are being recruited from three clinical sites in two Scottish National Health Service (NHS) Boards for 6 months. Eligible patients are all women who are: (1) aged between 18 and 65 years; (2) in paid employment or self-employed; (3) living or working in Lothian or Tayside, Scotland, UK; (4) diagnosed with an invasive breast cancer tumour; (5) treated first with surgery. Patients are randomly allocated to receive referral to a VR service or usual care, which involves no formal employment support. The primary outcome measure is self-reported sickness absence in the first 6 months following surgery. Secondary outcome measures include changes in quality of life (FACT-B), fatigue (FACIT-Fatigue) and employment status between baseline and 6- and 12-months post-surgery. A post-trial evaluation will be conducted to assess the acceptability of the intervention among participants and the feasibility of a larger, more definitive, trial with patients with lung and prostate cancer.

**Discussion:**

To our knowledge this is the first study to determine the feasibility of a randomised controlled trial of the effectiveness of VR services to enable people with cancer to remain in or return to employment. The study will provide evidence to assess the relevance and feasibility of a larger future trial involving patients with breast, prostate or lung cancer and inform the development of appropriate VR services for people living with cancer.

**Trial Registration:**

ISRCTN: ISRCTN29666484

Registration date: 07/10/10; Randomisation of first patient: 03/12/10

## 1 Background

Due to improvements in cancer survival the number of people of working age living with cancer across Europe is likely to increase [1]. It is estimated that more than 500,000 people in the UK under the age of 65 years have been diagnosed with cancer during their working lives [2]. Every year in Scotland around 27,400 people are diagnosed with cancer, 52% (n = 14,300) of whom are women [3]. Breast cancer is the most commonly diagnosed cancer in Scottish women and incidence has increased by 8% over the past decade attributed, in part, to earlier detection due to mammography screening programmes[3]. In 2007 over half (55%) of all women diagnosed with breast cancer in Scotland were of working age (i.e., between 18 and 65 years): around 1 in 20 were under 40 years, and 1 in 5 were under 50 years [4]. Despite notable variation across countries, [5] breast cancer mortality rates are decreasing across Europe[6,7]. In a recent comparative analysis of 30 European countries, England and Wales, Scotland, and Northern Ireland had the second, fourth and fifth largest overall decline in mortality between 1989 and 2006 of 35%, 30% and 29%, respectively [5]. There is evidence that the trends of increasing incidence [8] and decreasing mortality will continue [5]. Remaining in or returning to work will therefore be increasingly important for women living with breast cancer in Scotland and across the UK and Europe.

Dame Carol Black's recent review of the health of Britain's working age population recommended a multi-agency and partnership approach to supporting people to remain in or return to work and highlighted the role of line managers, general practitioners (GP) and vocational rehabilitation (VR) services[9]. UK governments have made commitments to reduce the number of working days lost to ill-health and to improve access to VR services. In Scotland, the Scottish Centre for Healthy Working Lives (SCHWL) has established pilot VR services in National Health Service (NHS) Tayside and NHS Lothian. These services support people to remain in or return to work by providing fast-track care and services such as physiotherapy, counselling and occupational therapy to employees from small- to medium-sized companies with less than 250 employees, where occupational health support services are not available. The Scottish Government has recently identified return to work for people living with cancer as a priority [10].

People with cancer can often experience changed workplace relations or employment status following diagnosis or during treatment, with negative financial and psychosocial consequences. It is known that individuals change jobs, leave paid employment or experience a decline in earnings [11]. Individuals may also experience discrimination from employers or colleagues [11]. However, a recent review has identified that there are few services to support people to remain in or return to work after cancer and no associated trials to assess their impact [11].

The SCHWL and Macmillan Cancer Support are developing plans to expand VR services to people with cancer. The establishment of pilot VR services across Scotland may provide an opportunity to conduct a large randomised controlled trial to evaluate their effectiveness for people living with cancer. However, there are several uncertainties that must be addressed including an assessment of the feasibility of such a trial and the acceptability of the intervention among people with cancer. Given increasing incidence and survival of people living with breast cancer, it is both timely and appropriate to determine the feasibility of this definitive trial through a pilot randomised controlled trial (RCT) of VR services among women following surgery for breast cancer. A study design incorporating a pilot RCT was selected to refine, and assess the feasibility of, trial processes (including, recruitment, randomisation and follow-up) and estimate the likely effect size in advance of a larger, more definitive, future trial.

## 2 Aim

The aim of this study is to determine the feasibility of an RCT of VR services among women with breast cancer following surgery and whether this intervention is acceptable in this patient group.

### 2.1 Objectives

1. Determine the numbers of patients in Lothian and Tayside with breast cancer referred for surgery who are in paid or self-employment and potentially eligible for the RCT.

2. Assess whether it is feasible and acceptable to recruit patients into the trial post-operatively. If not, identify the most appropriate timing for recruitment and the employment intervention that follows.

3. Estimate trial recruitment rates (i.e., percentage of eligible women who consent to the trial) and attrition over the period of the trial.

4. Determine whether the outcomes are measurable and appropriate, and evaluate the instruments used to measure secondary outcomes (i.e., FACT-B, FACIT-Fatigue).

5. Estimate the likely effect size to inform the power calculation for a larger, more definitive trial.

6. Assess the feasibility of including patients with lung and prostate cancer in a larger future RCT.

## 3 Methods

### 3.1 Design

**Figure 1:**
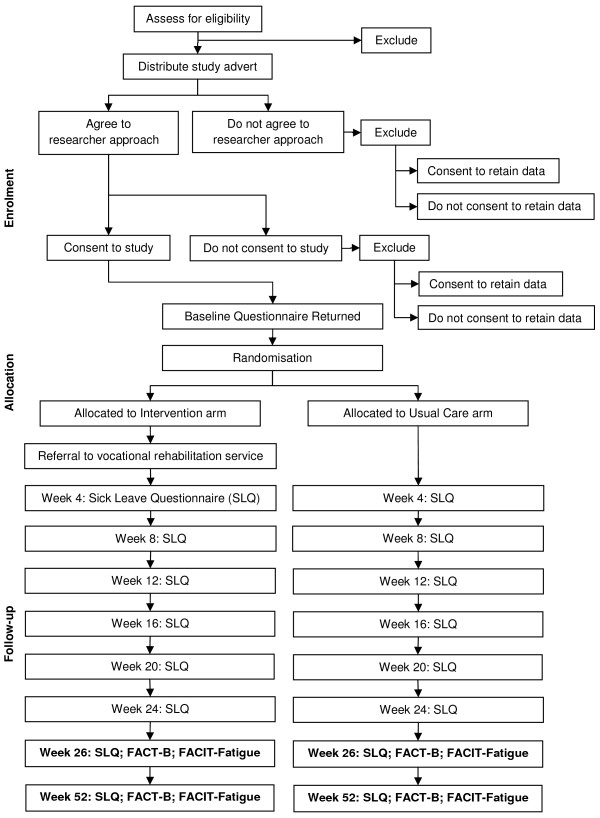
Study Design Flowchart: enrolment, randomisation and follow-up

The design of this feasibility study is an interventional two-arm RCT (Figure 1).

### 3.2 Setting and participants

Patients with breast cancer are being recruited from three hospitals in two NHS Boards in Scotland (Perth Royal Infirmary (PRI), and Ninewells Hospital, Dundee [NHS Tayside]; Western General Hospital (WGH), Edinburgh [NHS Lothian]). Recruitment to the trial is proceeding for 6 months at each site.

#### 3.2.1 Eligibility

Eligible patients are all women who are: (1) aged between 18 and 65 years; (2) in paid employment or self-employed; (3) living or working in Lothian or Tayside, Scotland, UK; (4) diagnosed with an invasive breast cancer tumour; (5) treated first with surgery. Patients diagnosed with Ductal Carcinoma in Situ (DCIS) are excluded. Patients are not excluded on the basis of communication difficulties. Interpretation and translation services will be used for participants unable to communicate in English. Patients with learning difficulties are also not excluded as a study investigator (GH) has expertise involving people with learning difficulties in research.

#### 3.2.2 Sample size calculation

There are several uncertainties that this feasibility study seeks to address before a larger, more definitive RCT of vocational rehabilitation services for people with cancer can be conducted. These uncertainties include: (1) the number of women who may be eligible for inclusion in the trial; (2) the percentage of eligible women who meet the VR services' criteria for referral; (3) the recruitment rate, defined as the percentage of eligible women who consent to involvement in the trial. The feasibility study will therefore provide evidence to inform sample size calculations for a larger trial. However, we estimate that between 66 and 79 patients will be recruited to the feasibility study over a 6 month period. This sample size is based on diagnosis and surgical data obtained from 2008, estimated employment rates, and a conservative recruitment rate of between 50% and 60%.

The feasibility study is also assessing the likely effect size to inform sample size calculations for a potential larger future trial. The primary outcome measure is the number of days off work in the 6 months following surgery. One report indicated that the mean length of time absent from work after a diagnosis of breast cancer was 5.6 months [12]. Assuming, therefore, a mean of 180 days absent from work in the control group and an estimated standard deviation of 100 days, with a sample size of 70 (35 in each group), the study would be able to detect a reduction in number of days absent from work from 180 to 110 days with 5% level of significance and 80% power. A larger sample size would be required to detect smaller differences. To detect a reduction in number of days absence by 2 months, a sample size of 88 patients (44 in each group) would be required. To detect a reduction of 1 month, a sample size of 350 (175 in each group) would be required.

### 3.3 Patient Recruitment

Recruitment and obtaining written informed consent to be involved in the study is occurring before participants are randomly assigned to the intervention or usual care arm of the study. Breast care nurses are initially applying the inclusion criteria to patients attending hospital in the pre-operative phase. Due to variation in local clinical processes, service size and workforce capacity the recruitment process differs in each of the three hospitals.

#### 3.3.1 Perth Royal Infirmary (PRI)

Women who are eligible for inclusion in the RCT are identified from surgical lists by clinical teams. Eligible women are given information about the study by their breast care nurse at a pre-operative appointment. Written informed consent is taken at a face-to-face meeting with a researcher (RGK) when women attend hospital for a post-operative clinic appointment.

#### 3.3.2 Ninewells Hospital, Dundee

Eligible women are identified from surgical lists by clinical teams and are given a pack of information about the study when they attend hospital for surgery. Women return this information pack to their breast care nurse when they leave hospital the following day or by post to a researcher (RGK). Those who agree to participate include their written informed consent in their returned pack.

#### 3.3.3 Western General Hospital (WGH), Edinburgh

Eligible women are identified by clinical teams from staging information discussed at a weekly multi-disciplinary team meeting. Eligible women are given a pack of study information by their breast care nurse when they attend hospital for a pre-operative appointment and return this to their breast care nurse when they attend for surgery or by post to a researcher (RGK). Those who agree to take part in the trial include their written informed consent in the returned information pack.

In each hospital, patients who do not want to participate are asked if they consent to the retention of a limited set of anonymised clinical, sociodemographic and employment data (e.g., age, sex, postcode, diagnosis, treatment, employment status) so that a comparison can be made between patients who do and do not agree to be approached (Figure 1). Data are recorded on the number of patients who are eligible and reasons for ineligibility, the numbers who consent to participate in the trial and the numbers who consent to retention of anonymised data.

### 3.4 Randomisation

The allocation sequence was generated from a Bernoulli probability distribution with a specified probability of 0.5 which ensures participants have an equal chance of being in either group. Participants are randomly assigned to the intervention and usual care arm with a 1:1 allocation ratio (Figure 1). A separate sequence is used for each NHS Board (analogous to the VR service to which participants in the intervention arm are referred) to ensure that there is an even distribution of participants to the intervention and usual care groups in each NHS Board. The allocation sequences are concealed from the researchers responsible for recruiting patients into the trial (RGK), data collection (RGK) and data analysis (RGK, JE, NMG, GH). The trial statistician (DA) provided the allocation sequences to an administrator in the Cancer Care Research Centre at the University of Stirling who is not involved in the process of random number generation nor data collection and analysis. This individual will assign patients to the intervention and usual care groups and refer patients in the intervention arm to the VR service.

#### 3.4.1 Blinding

It will be clear to most patients whether or not they have been assigned to the intervention or usual care arm of the study. Thus, it is not possible to blind patients to their group allocation. However, the randomisation procedure outlined above ensures that those involved in data collection and analysis will not know to which group patients have been randomly assigned.

### 3.5 Intervention

The complex intervention is referral to a VR service in either Tayside or Lothian (Figure 1). Patients recruited from PRI and Ninewells, Dundee are randomised to receive referral to a VR service in Tayside; individuals enrolled from WGH are referred to a VR service in Lothian. All participants allocated to the intervention arm of the trial are contacted by a VR service by telephone within 10 days following return of the baseline questionnaire to the researcher (RGK). Participants are allocated a 'case manager' who conducts a telephone assessment of supportive care needs to facilitate remaining in or returning to work during which baseline measures are also recorded using the Canadian Occupational Performance Measure (COPM) [13], 12 item General Health Questionnaire (GHQ-12) [14] and European Quality of Life - 5 Dimensions (EQ-5D) [15]. Outcomes are re-measured at 3- and 6-months from referral. Based on this initial assessment of each individual's personal goals and health status the case manager signposts participants to appropriate support services including physiotherapy, occupational therapy, occupational health nurse, occupational health doctor, counsellor/psychological therapy, complementary therapy. Each individual may therefore receive a different (combination of) intervention(s). Usual care following surgery involves no formal employment support. Participants in both arms of the trial receive a copy of the booklet *Work and Cancer *published by Macmillan Cancer Support [16].

As the personalised and complex nature of the intervention precludes exogenous standardisation, data will be obtained from each service on the specific interventions received by individuals in the intervention arm of the pilot RCT. Secondary analysis of outcome measures recorded by each service at initial assessment and 3- and 6-month follow up will also be undertaken for these women. In addition, information will be gathered about each service (e.g., size, workforce composition, ethos) to contextualise possible differences in reported outcomes between VR services.

### 3.6 Data collection

Data to evaluate the intervention in terms of quality of life, fatigue and change in employment status are collected at baseline, 6 months and 12 months after entry to the trial via a self-completion questionnaire. In addition, self-reported sickness absence is collected every four weeks for the first 6 months of follow-up and for the four weeks before the 12-month follow-up time point (Figure 1). A telephone reminder is administered if questionnaires are not returned within 10 days. Participants have the option of a researcher collecting their responses to all questionnaires by telephone.

### 3.7 Outcome measures

#### 3.7.1 Primary outcome

The primary outcome measure is self-reported number of days off work due to ill-health within the first six months after surgery.

There are two main ways of measuring absence from work: (1) sick leave register at participants' place of employment; (2) participant self-report. Although a sick leave register managed by the employer is regarded as the gold standard, self-report is also robust [17]. One study found more than 96% accuracy between register and self-report when the recall period is limited to 2 to 4 weeks [18]. The feasibility of using sick leave registers will be determined by asking participants to consent to contact with their employers to access their sick leave register at the end of the 6 month follow-up period.

#### 3.7.2 Secondary outcomes

Secondary outcome measures are changes in quality of life (QoL), fatigue and employment status.

##### 3.7.2.1 Quality of life

The Functional Assessment of Cancer Therapy-Breast Cancer (FACT-B) Version 4 will be used to assess breast cancer related QoL [19]. The FACT-B is a 37-item self-report questionnaire that evaluates several QoL domains: physical, social/family, emotional, and functional well being. In addition, a 10-item breast cancer subscale (BCS) is specific to the experiences of women living with breast cancer and the symptoms and side-effects of treatment and includes items on anxiety, pain and body image. Participants complete the questionnaire in terms of the past 7 days and each item is scored on a 5-point scale that varies from 0 (not at all) to 4 (very much). Negatively phrased questions are reversed prior to analysis and scores are summed for each domain and a higher score indicates higher well being [20]. Domain scores vary from 0 to 28 for the physical, social/family and functional well being domains; 0 to 24 for the emotional well being domain; and 0 to 40 for the BCS. The scores of the four well-being domains are summed to calculate the Functional Assessment of Cancer Therapy-General (FACT-G) score and total scores of the FACT-G and BCS are summed to calculate a FACT-B score.

##### 3.7.2.2 Fatigue

The Functional Assessment of Chronic Illness Therapy-Fatigue Scale (FACIT-Fatigue) is used to assess specific functional and physical aspects of fatigue associated with breast cancer diagnosis and treatment. This 13-item subscale has been determined to be a reliable and valid stand alone measure of fatigue [21]. In common with the FACT-B, items are scored on a 5-point likert scale and relate to the past 7 days. FACIT-Fatigue scores vary from 0 to 52 and negatively worded items are reversed before analysis so that higher scores represent better self-reported health [20].

##### 3.7.2.3 Employment status

A non-validated questionnaire will measure change in employment status in terms of five indicators: earnings, job, role, hours worked, or employment type (i.e., part-/full-time employment).

##### 3.7.2.4 Socio-demographic and clinical information

A structured questionnaire is used to collect data on date of birth, postcode, total household income, clinical diagnosis, and other conditions for which participants are currently receiving treatment. Consent will also be sought to access participants' hospital clinical notes to accurately identify diagnosis and treatments administered.

### 3.8 Data analysis

The study will provide data on eligibility, recruitment rates and possible loss to follow-up informing the feasibility and development of a larger, more definitive, future trial. The study will also assess the possibility of stratifying the sample in a larger trial by potential study confounders such as age, sex, employment type, hours of work, socio-economic status, co-morbidity and cancer stage that have previously been identified as impacting on length of absence from work [11].

As this is a feasibility study, hypothesis testing must proceed cautiously. However, it is anticipated that participants referred to VR services will experience fewer days off work due to sickness, lower levels of fatigue and increased QoL. QoL measures will be analysed at global (i.e., summed FACT-G and FACT-B scores), sub-scale (i.e., physical, social/family, emotional, functional, BCS), and individual question level. This will also enable evaluation of the outcome questionnaire's ability to detect differences in QoL between the intervention and control group associated with VR intervention. The greatest difference may be detectable at the level of individual domains of well-being (particularly, emotional, social/family and functional) and individual questions included in these domains (e.g., "I am satisfied with how I am coping with my illness" [GE2], "I feel close to my friends" [GS1], "I get support from my friends" [GS3], "I am able to work (include work at home)" [GF1], "My work (include work at home) is fulfilling" [GF2]) as support to remain in or return to employment may increase emotional well-being, enable access to a wider network of social support through colleagues, and increase physical functioning.

#### 3.8.1 Statistics

Data analysis will be conducted using PASW Statistics (Version 17) [22]. Baseline characteristics will be reported as mean and standard deviation for continuous data and n (%) for categorical data. Differences between the intervention and control groups for the primary outcome measure will be tested using an independent samples t-test. Differences between groups in secondary outcome measures will be examined by analysis of covariance adjusting for baseline values. Significance level is set at 0.05.

### 3.9 Post-trial evaluation

#### 3.9.1 Acceptability of the trial and VR intervention among trial participants

A patient-centred evaluation of the acceptability of the trial will be conducted by interviewing participants in each arm of the trial. Semi-structured telephone interviews will elicit participants' views on the acceptability of participating in the trial, the method and timing of recruitment and their opinions about approaching their employers directly. Maximum variation sampling will be used to initially identify 5 patients in each arm of the trial. Key variables in the sampling frame will include: current employment status (i.e., at/off work), job, role, tenure (i.e., part-/full-time), age, diagnosis, treatment pathway. Interviewing and analysis will proceed concurrently to pinpoint data saturation, and further rounds of interviews will be conducted with new participants, if required. The interviews will be digitally recorded and transcribed. NVivo (Version 8) [23] will support data management and qualitative analysis. Transcripts will be analysed thematically using the Framework approach [24], which is a method that provides a structure within which qualitative data are organised and in which themes are identified between participants and groups of participants. Data are processed through stages that familiarise the researcher with the content and enable indexing and coding. Thematic matrices are developed that preserve the richness of the data and enable data to be categorised and compared thematically. Data collection will be conducted by two study researchers (RGK, BC). Analysis will be conducted by RGK, BC, NMG, JE and GH.

#### 3.9.2 Feasibility of including patients with lung and prostate cancer in a future RCT

##### 3.9.2.1 Health Professional Interviews

Semi-structured face-to-face interviews will be conducted with purposively sampled staff from the lung and prostate cancer clinical teams (1 consultant and 1 Clinical Nurse Specialist (CNS) from each team [n = 4]), staff from the VR services in Tayside and Lothian (1 service manager and 1 case manager from each service [n = 4]). Interviews will elicit views on the acceptability of the pilot RCT (e.g., the method and timing of recruitment, eligibility criteria) and the feasibility of including patients with lung or prostate cancer in a future RCT.

##### 3.9.2.2 Patient Interviews

Face-to-face semi-structured interviews will also be conducted with people with either lung or prostate cancer. Individuals will be identified from clinical databases and recruited from the hospital at an appointment with their consultant. Interviews will elicit views on the acceptability of VR intervention and prospective trial participation, and explore employment issues that may have been encountered following diagnosis and treatment. Maximum variation sampling will be used to initially identify 5 patients with lung cancer and 5 people with prostate cancer. Key variables in the sampling frame will include: current employment status (i.e., at/off work), job, role, tenure (i.e., part-/full-time), age, diagnosis, treatment pathway. Individuals who were first treated with surgery will be approached to enable comparison with the group of women in the pilot RCT. Interviewing and analysis will proceed concurrently to pinpoint data saturation, and further rounds of interviews will be conducted with new participants, if required. The interviews will be recorded and transcribed and the transcripts analysed thematically using the Framework approach [24] supported by NVivo qualitative analysis software [23]. Data collection will be conducted by RGK and BC. Analysis will be conducted by RGK, BC, NMG, JE and GH.

### 3.10 Ethical considerations

The study protocol has been approved by NHS Tayside Committee on Medical Research Ethics A (Ref: 10/S1401/15) and the ethics committee of the University of Stirling. Research Governance approval was also obtained from NHS Lothian and NHS Tayside. Informed written consent is obtained from each participant. The participant information sheet provides contact details and a telephone number of the study's principal investigator (GH) and an independent contact to whom queries may be directed during the study period. With the participant's consent their GP and consultant is informed of their involvement in the study in accordance with good practice ethical guidelines.

#### 3.10.1 Data storage and confidentiality

All questionnaires, interview transcripts and consent forms will be stored securely in a locked filing cabinet. Quantitative and qualitative data are entered into password protected databases (PASW Statistics [Version 17] [22] and NVivo [Version 8] [23], respectively) in anonymised form and access is restricted to members of the research team. Following completion of the study, data will be archived and stored securely for 10 years in accordance with University of Stirling policy.

#### 3.10.2 Risks

The main known risk to participants is that those who are in the usual care arm of the study may feel aggrieved that they have not received support from a VR service. However, the participant information sheet explains clearly that there is a chance that individuals may not be randomly allocated to the intervention group. There is also the potential that all participants involved in the study may feel pressured to return to work because its focus is employment. However, the Macmillan Cancer Support booklet *Work and Cancer *that all participants receive makes it clear that patients should only remain in or return to work when they feel physically and mentally able to do so[16]. Study investigators will also reinforce this message in each encounter with participants.

## 4 Discussion

To our knowledge this is the first study to determine the feasibility of an RCT to assess the effectiveness of VR services to enable people with cancer to remain in or return to employment. The study will provide evidence to assess the relevance and feasibility of a larger future trial involving patients with breast, prostate or lung cancer. VR services may assist patients with cancer maintain their income, identity, and social networks, and improve quality of life potentially mitigating the negative financial and psychosocial consequences often associated with changes in employment status following cancer diagnosis or during treatment. Findings from this research will therefore inform the future development of appropriate VR services for people living with cancer, particularly for women with breast cancer following surgery.

## 5 Abbreviations

BCS: Breast Cancer Subscale; CNS: Clinical Nurse Specialist; COPM: Canadian Occupational Performance Measure; DCIS: Ductal Carcinoma in Situ; EQ-5D: European Quality of Life - 5 Dimensions; FACT-B: Functional Assessment of Cancer Therapy-Breast Cancer; FACIT-Fatigue: Functional Assessment of Chronic Illness Therapy-Fatigue Scale; FACT-G: Functional Assessment of Cancer Therapy-General; GHQ-12: 12 item General Health Questionnaire; NHS: National Health Service; PASW: Predictive Analytics SoftWare; PRI: Perth Royal Infirmary; SCHWL: Scottish Centre for Healthy Working Lives; VR: vocational rehabilitation; WGH: Western General Hospital, Edinburgh.

## 6 Competing interests

The authors declare that they have no competing interests.

## 7 Authors' Contributions

RGK is responsible for recruitment to the trial. RGK and BC will conduct data collection and analysis. JE and NMG contributed to the design of the study, secured funding, and will conduct data analysis. DA is the trial statistician and is responsible for generating the randomisation sequences and providing guidance on statistical analysis of trial data. GH designed the study, secured funding, is responsible for project oversight, and will conduct data analysis. RGK and GH wrote the first draft of the manuscript and BC, JE, NMG and DA commented on drafts. All authors approved the final version.
